# Hypothesis and Theory: Roles of Arginine Methylation in C9orf72-Mediated ALS and FTD

**DOI:** 10.3389/fncel.2021.633668

**Published:** 2021-03-23

**Authors:** Anna L. Gill, Alan S. Premasiri, Fernando G. Vieira

**Affiliations:** ALS Therapy Development Institute, Cambridge, MA, United States

**Keywords:** C9ORF72 ALS/FTD, dipeptide repeat, polyGR, polyPR, PRMT inhibitor, arginine methylation, splicing, chromatin remodeling

## Abstract

Hexanucleotide repeat expansion (G4C2_n_) mutations in the gene C9ORF72 account for approximately 30% of familial cases of amyotrophic lateral sclerosis (ALS) and frontotemporal dementia (FTD), as well as approximately 7% of sporadic cases of ALS. G4C2_n_ mutations are known to result in the production of five species of dipeptide repeat proteins (DRPs) through non-canonical translation processes. Arginine-enriched dipeptide repeat proteins, glycine-arginine (polyGR), and proline-arginine (polyPR) have been demonstrated to be cytotoxic and deleterious in multiple experimental systems. Recently, we and others have implicated methylation of polyGR/polyPR arginine residues in disease processes related to G4C2_n_ mutation-mediated neurodegeneration. We previously reported that inhibition of asymmetric dimethylation (ADMe) of arginine residues is protective in cell-based models of polyGR/polyPR cytotoxicity. These results are consistent with the idea that PRMT-mediated arginine methylation in the context of polyGR/polyPR exposure is harmful. However, it remains unclear why. Here we discuss the influence of arginine methylation on diverse cellular processes including liquid-liquid phase separation, chromatin remodeling, transcription, RNA processing, and RNA-binding protein localization, and we consider how methylation of polyGR/polyPR may disrupt processes essential for normal cellular function and survival.

## Introduction

A hexanucleotide repeat expansion (G4C2_n_) within the first intron of chromosome 9 open reading frame 72 (C9ORF72) represents the most common type of genetic mutation associated with increased risk of amyotrophic lateral sclerosis (ALS) and frontotemporal dementia (FTD; DeJesus-Hernandez et al., 2011; Renton et al., 2011). Three broad disease processes have been implicated: loss-of-function of C9ORF72, toxic gain-of-function of expanded sense and antisense RNA transcribed from the expanded repeat DNA region, and non-canonical translation of dipeptide repeat proteins (DRPs; Yang et al., 2020). Each of these domains has proved fertile territory for research with none being debunked and none being necessarily confirmed as primary drivers of neurodegeneration in humans harboring these mutations.

Compelling findings have arisen from the confirmation that DRPs are detectable in central nervous system tissues (CNS) of people with C9orf72 G4C2_n_-associated ALS/FTD (Ash et al., 2013; Cleary and Ranum, 2013; Gendron et al., 2013; Mori et al., 2013; Sakae et al., 2018). In particular, arginine-rich DRPs comprised of glycine-arginine (polyGR) or proline-arginine (polyPR), have consistently been demonstrated to be toxic in experimental systems (Freibaum and Taylor, 2017). Toxicity of polyGR/PR has been observed in cell culture systems (Mori et al., 2013; Kwon et al., 2014; Kramer et al., 2018), in *Drosophila* models (Mizielinska et al., 2014; Wen et al., 2014), and in mouse models (Zhang et al., 2018; 2019; Choi et al., 2019; Cook et al., 2020). We also demonstrated consistent and significant cell type and differentiation-specific toxicity of polyGR/PR in cell culture models (Gill et al., 2019).

**Figure 1:**
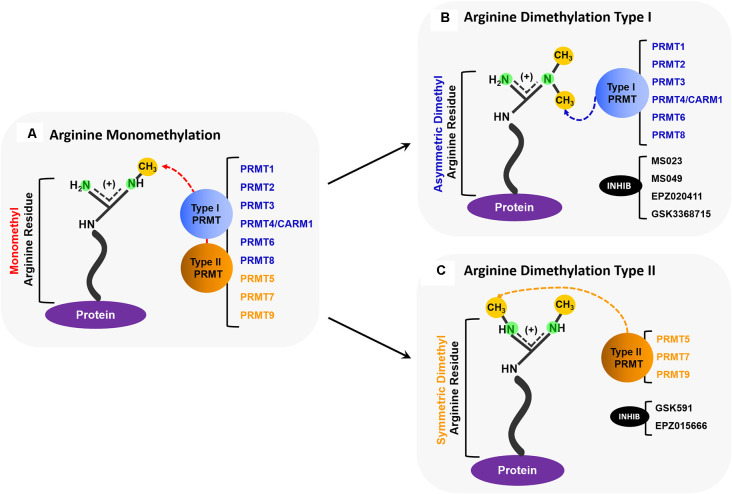
Overview of PRMT activity. **(A)** All PRMTs (PRMTs 1–9) can add one methyl group to a nitrogen atom on a protein arginine residue so that it becomes a monomethyl arginine (MMA). **(B)** Type I PRMTs (PRMTs 1, 2, 3, 4/CARM1, 6, and 8) can add an additional methyl group to the same nitrogen atom that was monomethylated to form an asymmetrically dimethylated arginine. Some small molecule inhibitors, such as MS023, MS049, EPZ020411, and GSK3368715, inhibit the activity of some Type I PRMTs. **(C)** Type II PRMTs (PRMTs 5, 7, and 9) can add a methyl group to the second nitrogen to form a symmetrically dimethylated arginine. Some small molecule inhibitors, such as GSK519, and EPZ015666, inhibit the activity of some Type II PRMTs.

We recently reported that the toxicity caused by exposure to polyGR/PR can be completely abrogated using inhibitors of Type I protein arginine methyltransferases (PRMTs; Premasiri et al., 2020). PRMTs are a family of enzymes responsible for the methylation of arginine residues in proteins. PRMT activity can result in monomethylation, symmetric dimethylation (SDMe), and asymmetric dimethylation (ADMe) of arginines (Figures 1A–C). Arginine methylation, sometimes erroneously conflated with DNA methylation, is one of the most extensive protein methylation reactions in mammalian cells (Paik et al., 2007). Arginine is the only amino acid with a guanidino group containing five potential hydrogen bond donors positioned favorably for interactions with biological hydrogen bond acceptors (Bedford and Clarke, 2009). The addition of methyl groups to arginine residues changes shape and removes potential hydrogen bond donors. This can sometimes result in changes that promote preferential inhibition of interactions with some, but not all, potential binding partners. In other cases, the methylation actually promotes interactions (Bedford and Clarke, 2009). Ultimately, these post-translational modifications have been demonstrated to be involved in many fundamental biological processes including transcription, RNA processing, DNA damage responses, and liquid-liquid phase separation (Guccione and Richard, 2019).

We explored the roles of PRMTs in polyGR/PR dependent toxicity based on the simple hypothesis that arginine-rich dipeptides would interact with enzymes evolved to modify arginine residues. Ample evidence exists suggesting the involvement of PRMTs in C9orf72 G4C2_n_-mediated ALS-FTD, and thus the involvement of arginine methylation as well. A *Drosophila* model employing ATG-mediated expression of polyGR/PR demonstrated colocalization of protein arginine methyltransferase 1 (PRMT1) with both species of dipeptides and showed that knocking down 4 out of 10 PRMTs can enhance PR toxicity (Boeynaems et al., 2016). Also, symmetrically dimethylated arginine (SDMe-R) in the cerebellum and hippocampus is markedly increased in people with C9ORF72-mediated neurodegeneration when compared to non-C9ORF72-mediated neurodegeneration (Chitiprolu et al., 2018). Furthermore, relative positive staining of symmetrically dimethylated arginine inclusions correlates with later onset of disease and slower disease progression in people with C9orf72 G4C2_n_ mediated ALS (Gittings et al., 2020). Also, asymmetrically dimethylated arginine (ADMe-R) has been detected in the cortex of people with C9orf72 G4C2_n_ mediated neurodegeneration (Sakae et al., 2018).

While our recent findings and various clinical histopathological relationships point to PRMT activity and arginine methylation states as disease-modifying in C9orf72 ALS/FTD, it remains very unclear how. In this article, we discuss the implications of arginine methylation in the context of the evidence of biological processes that have been observed in C9orf72-mediated neurodegeneration. We extend the discussion to offer hypotheses for which biological processes may be engaged when Type I PRMT inhibition is protective against toxicity caused by polyGR and polyPR exposure.

## Physicochemical Effects of Arginine Methylation

### Physicochemical Effects of Arginine Methylation on RNA Binding and Protein: RNA-Dependent Liquid-Liquid Phase Separation

RNA binding proteins (RBPs) bind RNA to form complexes that are responsible for a diverse set of post-transcriptional regulation mechanisms (Gerstberger et al., 2014; Corley et al., 2020). The specific protein, RNA molecule, and type of binding interaction are factors that ultimately determine the function of the protein-RNA complex (Rissland, 2017). With the discovery of ALS-linked mutations of some RBPs, the roles of protein-RNA complexes have been explored in the context of neurodegeneration. Specifically, protein-RNA liquid-liquid phase separation (LLPS) and the formation of membraneless organelles are functions that have been investigated by multiple groups (Zhao et al., 2018; Pakravan et al., 2020). The low-complexity domains (LCDs) or intrinsically disordered regions (IDRs) of RBPs have been points of focus due to their facilitation of LLPS (Corley et al., 2020; Yoshizawa et al., 2020).

The LCD regions of some RBPs contain RGG/RG motifs, which can act as determinants for RNA binding and influence LLPS of the RBP because of their physicochemical properties (Calnan et al., 1991; Lin et al., 2015; Chong et al., 2018). Specifically, the binding of arginine-rich motifs to RNA is mediated by the frequency of the repeat motif and some variation of hydrogen bonds, hydrophobic interactions, and aromatic pi-pi stacking (Chong et al., 2018; Hofweber and Dormann, 2019; Corley et al., 2020). Though the presence of RNA is not always required for phase separation to occur, some RBPs have been shown to have enhanced phase separation in the presence of RNA and, as previously mentioned, the specific RNA molecules involved can influence LLPS (Schwartz et al., 2013; Chong et al., 2018). Though the interactions between RBPs and RNA are often dynamic, there is evidence that RNA conforming to G-quadruplex or other base-exposing structures can allow for increased RBP recognition and interaction (Thandapani et al., 2013; Chong et al., 2018; Hentze et al., 2018). It was recently shown that RNA conformation, specifically G-quadruplex or not, can lead to the formation of either fractal networks or liquid-like droplets, respectively (Boeynaems et al., 2019). Given the arginine-rich makeup of polyGR and polyPR, there is reason to suspect that interactions with RNA will vary depending on their type of RNA-bonding interaction and peptide length, ultimately determining what product will result from their LLPS.

**Figure 2:**
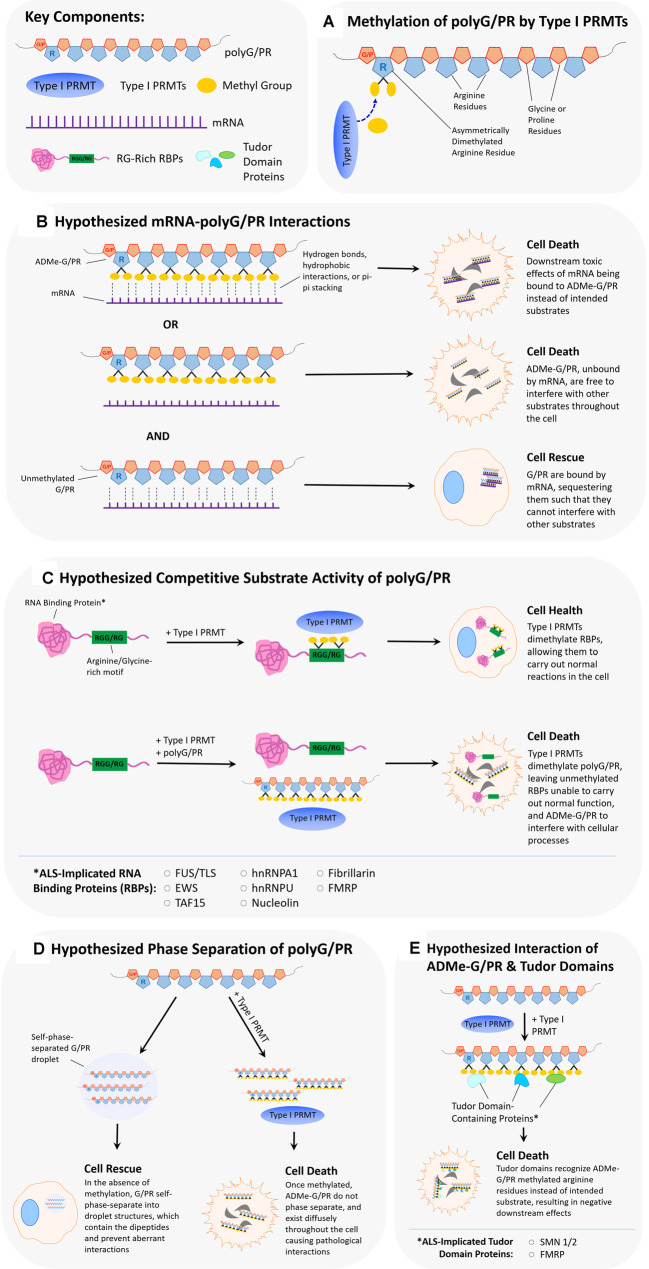
Hypothesized scenarios of how arginine dimethylation of polyG/PR affects their interactions with other biomolecules.** (A)** Asymmetrical dimethylation of the arginine residue of polyG/PR. **(B)** It is unclear whether methylation of arginine in polyG/PR increases or decreases their propensities to bind to RNAs. In one scenario, arginine dimethylation of polyG/PR may enable binding with RNA through hydrogen bonds, hydrophobic interactions, or pi-pi stacking, leading to downstream cellular toxicity as a result of aberrantly bound RNA. In another scenario, arginine dimethylation of polyG/PR may disrupt binding with RNA, leading to promiscuous interactions with other molecules that may result in cellular toxicity. Toxic effects would possibly be rescued or prevented by the binding and sequestration of polyG/PR by RNA. **(C)** PolyG/PR may function as competitive substrates of Type I PRMTs. The degree to which G/PR binds PRMTs will depend on their affinity to specific PRMTs and will ultimately influence the methylation-based activity of ALS-associated RNA binding proteins (RBPs) with RG/RGG motifs. **(D)** If unmethylated GR/PR self-phase separates in cells, they may be prevented from disrupting other cellular processes. Once arginine dimethylated, GR/PR may have more promiscuous cellular interactions resulting in cellular toxicity. **(E)** Arginine methylation of GR/PR could enable a diverse set of protein:protein interactions. Tudor domain-containing proteins, in particular, can specifically recognize methylated arginine residues, and methylated G/PR may interrupt normal processes that Tudor domain-containing proteins are involved in.

Arginine methylation of the RGG/RG motif can regulate the binding of RBP to RNA, and thus the LLPS of RBP-RNA complexes. The arginine methylation of the motif doesn’t affect its overall charge; however, it does change the distribution of charge and therefore the type of bonding interactions favored with RNA (Thandapani et al., 2013; Chong et al., 2018). Chong et al. (2018) summarize work from multiple labs conducted on heterogeneous nuclear ribonucleoprotein (RNP) A1 (hnRNPA1), Fragile X mental retardation protein (FMRP), heterogeneous nuclear ribonucleoprotein K (hnRNPK), and Ewing sarcoma (EWS), providing *in vitro* evidence that arginine methylation can either inhibit or have no effect on RNA binding. Conversely, a study looking at pancreatic cancer cells found that the arginine methylation of heat shock protein 70 (HSP70) enhanced its binding to B-cell lymphoma 2 (BCL2) mRNA, despite not containing an RGG/RG repeat motif (Wang et al., 2020). Thus, a protein with a methylated arginine motif can have enhanced RNA binding, but the surrounding amino acids can influence the effect. The effect of arginine methylation on protein-RNA granule formation is diverse as well. The RBP Ras GTPase-activating protein-binding protein 1 (G3BP1) is known to form RNA stress granule and this process is regulated by the methylation of its RGG motif. A study looking both at PRMT1 and PRMT5 overexpression and dimethylation activity demonstrated that arginine methylated G3BP1 was less able to form stress granules, and this suppressed activity was relieved upon demethylation (Tsai et al., 2016). On the other end of the spectrum, RNA-associated protein 55 (RAP55A) was shown to require arginine methylation of its RGG motif to properly form P-bodies (Matsumoto et al., 2012). Beyond the study of granule and ribonucleoprotein (RNP) dynamics, there has been work looking at RBP binding to non-coding RNA. Specifically, PRMT1 methylation of the RGG domain in translocated-in-sarcoma (FUS/TLS) inhibited its binding to long non-coding RNA (Cui et al., 2018). Arginine methylation of an RBP can lead to multiple effects on protein-RNA granule formation and overall RNA binding capability. The arginine methylation of polyGR or polyPR may allow them to associate with certain RNA structures aberrantly, leading to toxic downstream effects (Figures 2A,B). Alternatively, arginine methylation of these DRPs may suppress their binding to RNA, where binding would lead to sequestration and reduced toxic effects. There is evidence suggesting the glycines in RGG/RG motifs are responsible for the conformational flexibility involved when binding to RNA, leading to the possibility of polyGR having different binding interactions than polyPR, further influenced by each peptide’s methylation state (Chong et al., 2018; Hentze et al., 2018). It is important to note the work surrounding the C9orf72 expansion mutation RNA, which has been shown to form G-quadruplexes and also be involved in phase separation (Conlon et al., 2016; Fay et al., 2017). Given the nature of polyGR and polyPR, these two hallmarks of C9-associated ALS may be involved in a joint mechanism regulated by arginine methylation.

In short, given their similarities to RNA-binding motifs of many RNA-binding proteins, the presence of polyGR and/or polyPR may disrupt cellular homeostasis by binding directly to RNA and affecting RNA metabolism or translation (Figure 2). Like with RBP RNA-binding motifs, this proposed association between arginine-rich DRPs and RNA molecules might be influenced by arginine methylation states. Inhibition of type I PRMTs may decrease associations of polyGR and polyPR with RNA molecules in the cell. Alternatively, the presence of polyGR and polyPR in the cell may disrupt the normal post-translational arginine methylation of RBPs by PRMTs by acting as a competitive sink (Figure 2C). This may affect normal RBP/RNA interactions and cause harm to cells. Inhibition of Type I PRMTs may return the cell to near-homeostatic conditions.

### Physicochemical Effects of Arginine Methylation on Protein Only LLPS

While more research to date has focused on phase separation involving protein-RNA complexes, there has been work demonstrating that proteins with arginine-rich motifs undergo LLPS spontaneously. *In vitro*, purified proteins such as the RNA helicase LAF-1, DEAD-box helicase 4 (Ddx4), and FMRP will phase separate without the presence of RNA and will not phase separate with the deletion of RGG motifs (Elbaum-Garfinkle et al., 2015; Nott et al., 2015; Tsang et al., 2019). Similarly, synthetic glycine-arginine 20 mers (GR20) and proline-arginine 20 mers (PR20) also phase separate *in vitro* in the presence of the crowding agent PEG (Boeynaems et al., 2017). Furthermore, a comparison of the ability of various other proteins with arginine-rich motifs to phase separate determined that the phase separation was positively correlated to its proportion of arginine residues and peptide length (Boeynaems et al., 2017). The effect of arginine methylation on LLPS of RGG/RG-containing proteins so far is decidedly suppressive (Hofweber et al., 2018). *In vitro*, the arginine methylation of FUS, Ddx4, and heterogeneous nuclear ribonucleoprotein A2 (hnRNPA2) by PRMT1 destabilized their ability to form phase-separated droplets (Nott et al., 2015; Hofweber et al., 2018; Ryan et al., 2018). Of particular relevance is a recent study demonstrating that either ADMe or SDMe of GR20 reduced its ability to phase separate (Gittings et al., 2020). Given our findings demonstrating abrogated toxicity of polyGR and polyGR when Type I PRMTs are inhibited (Premasiri et al., 2020), it is plausible that reduced ADMe of polyGR and polyPR allows for a greater degree of phase separation. However, when these DRPs are asymmetrically dimethylated by Type I PRMTs, their propensity to phase separate decreases, and the scope of possible aberrant interactions increases (Figure 2D).

### Physicochemical Effects of Arginine Methylation on Protein:Protein Interactions

Just as the biochemical properties of RGG/RG motifs may drive RBP interactions with RNA, the RGG/RG motifs can be key determinants in various protein: protein interactions (Thandapani et al., 2013). Examples of this include hnRNPA1 association with HIV protein Rev, EWS self-association, and nucleolin association with hepatitis C virus protein NS5B, though the effects of arginine dimethylation in these contexts remain incompletely explored (Kusakawa et al., 2007; Hadian et al., 2009; Shaw et al., 2010; Thandapani et al., 2013). One example of arginine methylation directly disrupting a well-characterized protein:protein interaction is between FUS and the nuclear import receptor Transportin (TRN). The FUS C-terminus RGG motif is a binding site for TRN, and the arginine methylation of the motif impairs the association of FUS and TRN, in turn affecting the nuclear localization of FUS (Dormann et al., 2012). Arginine methylation can also indirectly influence protein:protein interactions. For example, PRMT1 methylation of FUS prevents binding with coactivators CREB-binding protein and p300 (CBP/p300; Cui et al., 2018), even though the FUS association with CBP/p300 occurs at the FUS N-terminus, where there is no arginine methylation site (Wang et al., 2008). This suggests that FUS arginine methylation by PRMT1 can allosterically alter FUS/TLS confirmation that its interaction with CBP/p300 is prevented.

Arginine methylation may also promote specific protein:protein interactions. For example, the survival of motor neuron (SMN) protein exhibits enhanced association with small nuclear ribonucleoproteins (snRNPs), SmD1, and SmD3, when arginine methylation is uninhibited (Friesen et al., 2001a). Like SMN, other proteins containing Tudor domains can bind specifically to methylated arginines through pi bonding and hydrophobic interactions (Fuhrmann et al., 2015). SnRNPs have been demonstrated to bind to polyGR and polyPR (Yin et al., 2017). The dynamics of this association may be heavily influenced by arginine methylation states of the dipeptide repeat proteins. Although methylation of polyGR and polyPR have been observed in experimental models (Boeynaems et al., 2016) and in post-mortem ALS central nervous system tissue (Gittings et al., 2020), the exact nature of any interactions between specific PRMTs and polyGR or polyPR remain uncharacterized. However, PRMTs have a higher affinity for arginine residues flanked by glycines because of greater conformational flexibility (Fuhrmann et al., 2015). This same conformational flexibility may play roles in the differences between polyGR and polyPR physicochemical interactions, as changes in charge distribution or conformational malleability due to arginine methylation may not be as pronounced for the proline-enriched polyPR. Regarding non-LLPS protein:protein interactions, we suspect asymmetrically dimethylated polyGR and polyPR could have enhanced interactions with other proteins, such as Tudor domain-containing molecules (Figure 2E). Our results demonstrated a complete abrogation of polyGR toxicity with Type I PRMT inhibitors, and near-complete abrogation of polyPR toxicity (Premasiri et al., 2020). This small difference may be attributable to additional conformational flexibility conferred on methylated polyGR, leading to a different cellular interactome.

## Effects of Methylating Arginine on Membraneless Organelles

### Stress Granules

**Figure 3:**
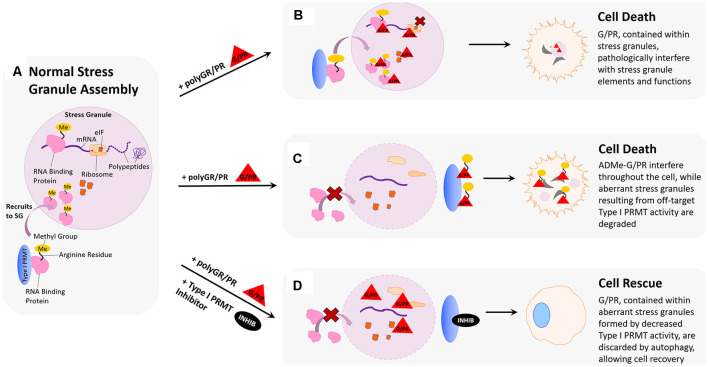
Hypothesized impact of polyGR/PR on stress granules and rescue by PRMT inhibition. **(A)** Normal stress granule assembly: in response to stress, the cell forms a membrane-less stress granule (SG) that contains important translational machinery crucial to cell survival, such as ribosomal subunits, elongation-initiation factors (eIFs), and RNA binding proteins (RBPs). Methylation of arginine residues on RNA binding proteins results in their recruitment to stress granules, where they can regulate translation to cope with cell stress. **(B)** Pathological scenario 1: GR and PR localize to stress granules. Methylation of RBPs results in their recruitment to stress granules where harmful interactions with polyGR/PR are facilitated by increased proximity. **(C)** Pathological scenario 2: GR and PR could act as substrates for type I PRMTs. This could result in competitive inhibition of appropriate RBP methylation, thereby inhibiting protective recruitment to stress granules and impeding stress responses. In a feed-forward scenario, methylated polyG/PR are less predisposed to self-phase separate and may interfere with additional cellular processes. **(D)** Rescue scenario: PRMT inhibition would reduce recruitment of RBPs to stress granules, thereby reducing the frequency of pathological RBP interactions with polyG/PR. This reduction of RBP stress granule recruitment could also result in the formation of aberrant stress granules containing G/PR, which are recognized by the cell as abnormal and thus degraded. Degradation of stress granules containing G/PR during the period of type I PRMT inhibition could potentially clear enough G/PR from the cell to facilitate cell recovery.

Cells form stress granules in response to conditions such as glucose starvation, heat shock, oxidative stress, and energy deprivation (De Leeuw et al., 2007; Fujimura et al., 2009; Groušl et al., 2009). These large, complex ribonucleoprotein particles contain messenger RNA (mRNA), eukaryotic initiation factors (eIFs), RNA-binding proteins (RBPs), and small ribosomal subunits, making them crucial for RNA processing during cellular stress (Figure 3A; Xie and Denman, 2011). Interestingly, much of the stress granule formation, composition, and sequestration is influenced by methylation of proteins harboring arginine-rich motifs, particularly those where arginine residues are flanked by glycines, such as RG or RGG. For example, the ALS-implicated protein FUS contains three arginine/glycine-rich regions and its propensity to localize with stress granules can be modulated by conditional the overexpression of PRMT1 in HEK293 cells (Yamaguchi and Kitajo, 2012; Ozdilek et al., 2017). In the context of multiple ALS-linked FUS mutations, depletion of PRMT1 in HEK293 cells *via* siRNA knockdown diminishes detergent-insoluble cytoplasmic inclusions of mutant FUS and related provocation of stress granules (Tradewell et al., 2012). Other neurodegeneration-associated proteins have been linked to this phenomenon, such as FMRP which contains an RG-rich region that can be methylated *in vitro* (Eichler et al., 1993; An et al., 2004; Denman et al., 2004). One immunostaining study has shown that endogenous FMRP forms small cytoplasmic granules in cultured cells and that after treating HeLa cells with a general methylation inhibitor adenosine dialdehyde (AdOx), the number of these cytoplasmic FMRP granules increased (Dolzhanskaya et al., 2006). Another study has even suggested that ALS-related protein C9orf72 and autophagy receptor p62 associate with stress granules harboring symmetrically dimethylated arginine motifs, and that symmetric dimethylation by PRMT5 drives the formation of a complex that eliminates stress granules by autophagy (Chitiprolu et al., 2018). Further, arginine methylation of RG-rich motifs within other proteins, including DNA topoisomerase 3B (TOP3B; Huang et al., 2018), hnRNPA1 (Wall and Lewis, 2017), eukaryotic initiation factor 2A (eIF2α; Haghandish et al., 2019), and ubiquitin-associated protein 2-like (UBAP2L; Huang et al., 2020), can influence stress granule formation and localization.

Given that methylation of RG-rich motifs influences stress granule formation and composition, it is important to consider how the introduction of arginine-rich dipeptide repeat proteins, polyGR, and polyPR, might influence stress granule biology in the context of C9orf72-mediated ALS. As previously discussed, the RGG-rich nature of stress granule proteins facilitates the incorporation of RNA and poly(ADP-ribose) and contributes to LLPS and aggregation of prion-like domains (PrLDs; Schwartz et al., 2013; Altmeyer et al., 2015; Burke et al., 2015; Lin et al., 2015; Molliex et al., 2015; Patel et al., 2015; Aguzzi and Altmeyer, 2016). Similarly, the arginine-rich nature of many ALS-associated mutant proteins confers upon them these properties and more, including protein binding promiscuity (Dormann et al., 2012; Thandapani et al., 2013). Further, it has been demonstrated that arginine-rich GR20 and PR20 are strongly phase-separate in the presence of a molecular crowder and that the extent of this phase separation is linked to arginine content, as arginine-lacking DRP GP20 did not exhibit phase separation (Boeynaems et al., 2017). As phase separation has been shown to promote stress granule assembly and drive pathological fibrillization (Molliex et al., 2015), LLPS of GR20 and PR20 raises the possibility that polyGR and polyPR might promote stress granule assembly in C9-ALS cells, though evidence of this effect is inconsistent; indeed, sometimes contradictory (Kwon et al., 2014; Wen et al., 2014; Tao et al., 2015; Lee et al., 2016). Another study demonstrated that overexpression of a codon-optimized PR_100_ construct in HeLa cells led to accrual of cytoplasmic PR_100_ granules that were positive for stress granule markers (Boeynaems et al., 2017). Similarly, overexpression of GFP-GR_100_ in HEK293T cells induced the formation of stress granules and delayed their disassembly as compared to HEK293T cells overexpressing GFP alone (Zhang et al., 2018). A recent study demonstrated that expression of polyGR and polyPR in transfected HeLa cells induced stress granule formation, with polyGR and polyPR expression causing stress granules to form in 45% of cells and 24% of cells, respectively (Sun et al., 2020).

The effects of polyGR and polyPR on phase separation and stress granule dynamics raise the question of whether such interactions contribute to the cytotoxic effects of the dipeptides (Figure 3B). If toxic polyGR and polyPR affect stress granule assembly and function, it may be true that modulating stress granule assembly in the presence of these mutant dipeptides could be therapeutic. As previously discussed, protein arginine methylation activity alters stress granule formation, with several studies emphasizing significant changes upon modulating the expression of the type I PRMT, PRMT1 (Tradewell et al., 2012; Yamaguchi and Kitajo, 2012). We have demonstrated that small molecule type I PRMT inhibitors dose-dependently abrogate cytotoxicity associated with 24 h polyGR or polyPR challenge in mouse spinal cord neuroblastoma hybrid cells (NSC-34) cells (Premasiri et al., 2020). The rescue effect we observe may be because type I PRMT inhibition alters the arginine methylation associated with stress granule formation and that the resulting changes in stress granule assembly, composition, and dynamics are beneficial to the cell when in the presence of polyGR and polyPR-induced stress (Figure 3D). For example, aberrant stress granule dynamics resulting from pathological protein aggregation have been shown to lead to stress granule degradation *via* autophagy, which could contribute to rapid degradation of polyGR and polyPR and cell rescue (Verdile et al., 2019). This hypothesis is bolstered by evidence that autophagic clearance of stress granules is protective in human induced pluripotent stem cell (iPSC) neuronal and astrocytic models of mutant SOD1 and TDP43 mediated ALS cell death (Barmada et al., 2014).

Still, the rescue effect we observe upon administering type I PRMT inhibitors could also suggest that polyGR and polyPR may be direct substrates for PRMTs. Given that the toxic DRPs are arginine-rich, in particular with polyGR acting as a repeat “RG” motif that is often associated with stress granule proteins, it is feasible that arginine residues within polyGR and polyPR could be methylated by PRMTs (Figure 3C). This possibility is supported by results from our study, where, following *in vitro* incubation with PRMT1 and methyl donor S-adenosyl methionine (SAM), polyGR is detectable by an antibody raised against asymmetrically dimethylated arginine 3 in Histone 4 (H4R3me2a; Premasiri et al., 2020). If polyGR and polyPR are substrates for type I PRMTs, and we observe that inhibiting type I PRMTs diminishes cytotoxic effects, this could suggest enhanced toxicity of polyGR and polyPR following their asymmetric arginine dimethylation contributes to their pathology. This possibility is also supported by results in our study, where NSC-34 cells challenged with synthetic GR_15_ or PR_15_ and NSC-34 cells challenged with synthetic ADMe-GR_15_ or ADMe-PR_15_ were compared. In these experiments, we observed that ADMe-GR_15_ and ADMe-PR_15_ dose-dependently induced more dysmetabolism and cytotoxicity than GR_15_ or PR_15_ (Premasiri et al., 2020). Such an increase in DRP toxicity with methylation could imply numerous underlying biological effects, one being that the methylation state of polyGR and polyPR could influence their localization in the cell concerning stress granules. If polyGR and polyPR have a propensity to undergo LLPS and form self-associated puncta in their unmethylated state, methylation of these proteins could permit them to exist more diffusely throughout the cell, free to interact with other proteins.

These two plausible mechanisms for how polyGR and polyPR affect stress granule composition and dynamics could exist simultaneously. If polyGR and polyPR are substrates for type I PRMTs, their presence may shift type I PRMT activity away from methylating key stress granule proteins, and instead towards methylation of aberrant polyGR and polyPR. As a result, stress granule proteins may be left unmethylated, or even methylated differently, perhaps symmetrically dimethylated by type II PRMTs. These changes in methylation state for stress granule proteins could result in pathological stress granule dynamics and composition. This could then be compounded by the increased toxicity of methylated polyGR and polyPR. In summary, promoting pathologically altered methylation patterns for key stress granule proteins, and altering the cellular distribution of methylated polyGR and polyPR in a way that favors pathological interaction with other proteins essential for cellular homeostasis, could both contribute to arginine-rich DRP pathogenesis, either additively or synergistically.

### Chromatin Remodeling

**Figure 4:**
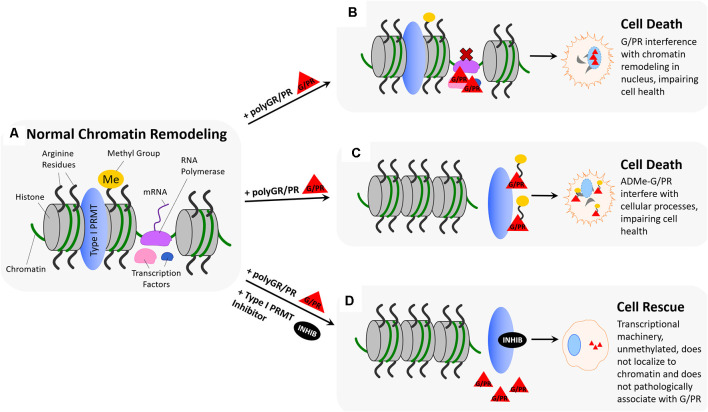
Hypothesized impact of polyGR/PR on chromatin dynamics and rescue by PRMT inhibition. **(A)** Normal chromatin remodeling: PRMTs methylate arginine residues on histones H3 and H4 resulting in separation of histones and recruitment of RNA polymerase (RNA Pol.), initiating production of immature mRNA from unwound chromatin. **(B)** Pathological scenario 1: RNA polymerase and transcription factors recruited to chromatin by PRMT methylation of histone arginine residues result in pathological protein:protein interactions between G/PR and transcriptional regulators. As a result, transcription proceeds irregularly or is unable to proceed. **(C)** Pathological scenario 2: GR/PR act as substrates for Type I PRMTs, resulting in methylation of GR/PR at chromatin instead of histone arginine residue methylation. Non-methylated histones remain tightly coiled, and ADMe-GR/PR are released to interfere with cellular proteins throughout the nucleus and cytoplasm. **(D)** Rescue scenario: With the introduction of a type I PRMT inhibitors such as MS023 or GSK3368715, Type I PRMT activity is inhibited, preventing histone arginine residue methylation. As a result, histones remain tightly coiled, and no transcriptional regulators are recruited to chromatin, preventing pathological GR/PR interactions with those proteins. Further, no type I PRMTs methylate GR/PR, which in their unmethylated form, have a propensity to form droplets, potentially preventing them from interfering with cellular proteins.

Chromatin remodeling refers to changes in chromatin structure that occur during regulatory processes and is generally defined as any event that alters the nuclease sensitivity of a chromatin region (Figure 4A; Aalfs and Kingston, 2000). Chromatin remodeling is essential for transcriptional activation, which is modulated by the p160 family of coactivators (SRC-1, GRIP1/TIF2, and p/CIP) at nuclear receptors (Sheppard et al., 1999; Belandia and Parker, 2000). Methylation of arginine residues on histones by PRMTs influences the activity of these coactivators (Chen et al., 1999; Schurter et al., 2001; Stallcup, 2001; Strahl et al., 2001; Wang et al., 2001; An et al., 2004; Lee et al., 2005; Pal and Sif, 2007). In this body of research, there is consensus that PRMTs 1 and 4 act as secondary coactivators alongside the p160 coactivator complex by methylating arginine residues on histones H3 and H4. Another type I PRMT, PRMT6, has been implicated in the regulation of DNA polymerase beta to affect DNA base excision repair (El-Andaloussi et al., 2006), and in methylation of H3R2 to negatively regulate N-terminal H3 tail binding (Iberg et al., 2008). The symmetric arginine dimethylation activity of type II PRMT, PRMT5, has also been implicated in both ATP-dependent chromatin remodeling required for myogenesis (Dacwag et al., 2009), and histone 4 arginine methylation that controls neural stem cell proliferation and differentiation (Chittka et al., 2012). Many of these processes have been implicated in neurodegenerative and neuromuscular diseases. For example, PRMT6 has been shown to enhance polyglutamine-expanded androgen receptor function and toxicity in spinal (SMA) and bulbar (SBMA) muscular atrophy (Scaramuzzino et al., 2015). Additionally, numerous PRMTs have been shown to regulate skeletal muscle plasticity, and show signs of dysregulation in neuromuscular disorders such as Duchenne muscular dystrophy (DMD), SMA, and ALS (Stouth et al., 2017). Similarly, PRMT5 symmetric dimethylation activity is attenuated by mutant huntingtin in Huntington’s disease (HD), suggesting that its deficiency is playing a role in HD pathogenesis (Ratovitski et al., 2015).

These studies suggest that methylation of arginine residues on histones and histone-associated proteins influences integral chromatin remodeling processes, including transcriptional activation and DNA damage repair. PolyGR and polyPR have also been shown to influence chromatin remodeling. Some reports suggest that polyPR and polyGR localize to and bind to nucleoli, altering rRNA transcription and processing (Kwon et al., 2014), while others demonstrate that polyGR and polyPR-induced reductions in transcription and rRNA maturation ultimately lead to nucleolar stress (Haeusler et al., 2014). PolyGR- and polyPR-associated loss in nucleolar integrity has been demonstrated in cell lines derived from C9-ALS patients by imaging the migration and localization of nucleolar proteins, including nucleolin and nucleophosmin, which are crucial for chromatin remodeling (Haeusler et al., 2014; Kwon et al., 2014). Aggregation of polyPR in nucleoli has been shown to increase nucleolar size, and disperse nucleolin in rat primary cortical neurons and human iPSC-derived neurons (Wen et al., 2014). Similarly, polyGR interaction with nucleoli has been shown to lead to nucleolar swelling, nucleophosmin translocation to the nucleus, and reduced levels of 18S rRNA and 28S rRNA in cellular and *Drosophila* models of ALS as well as in ALS patient tissue (Kwon et al., 2014; Mizielinska et al., 2014; Tao et al., 2015). These impacts of polyGR and polyPR on nucleolar function have consequences on chromatin remodeling and subsequent transcription, as chromatin housed within the nucleolus gives rise to over 60% of the entire RNA pool (Schöfer and Weipoltshammer, 2018). Nucleocytoplasmic transport and the Notch signaling pathway both influence chromatin remodeling as well, and be impaired in the presence of polyGR and polyPR (Jovičić et al., 2015; Yang D. et al., 2015). Most recently, polyGR and polyPR have been shown to inhibit homology-directed DNA double-strand break repair (Andrade et al., 2020), and to disrupt karyopherin-mediated nuclear import (Hayes et al., 2020).

Taken together, the evidence demonstrating that chromatin remodeling is closely linked to arginine methylation of histones by PRMTs, and the evidence showing cytotoxic effects associated with nucleolar localization of polyGR and polyPR, may again link untoward effects of polyGR/PR with PRMT activity. As previously discussed, type I PRMT inhibitors are capable of dose-dependently abrogating cytotoxicity associated with 24 h polyGR or polyPR challenge in NSC-34 cells (Premasiri et al., 2020). One possible explanation for this observed rescue effect could be that inhibiting type I PRMT activity limits methylation of arginine residues on histones H3 and H4 by PRMT1 and PRMT4, thus limiting transcriptional activation in a way that proves beneficial in the presence of aberrant polyGR and polyPR (Figure 4D). This idea is supported by evidence that histones and other chromatin-associated proteins are responders to cellular stress and are noted to change in function and localization according to methylation state (Smith and Workman, 2012; Weiner et al., 2012). Similarly, it may be the case that methylation of arginine residues on aberrant polyGR and polyPR influences their localization, potentially being determinant for their disruptive recruitment to chromatin (Figure 4B). Further, it is conceivable that limiting PRMT-mediated histone methylation activity could, in turn, limit crucial transcription factors and proteins, such as RNA polymerase II, from being recruited to chromatin where nucleolar polyGR and polyPR could begin pathogenic protein:protein interactions. Alternatively, type I PRMT inhibition could result in compensatory upregulation of type II PRMT activity, resulting in histone arginine residues being symmetrically dimethylated instead of asymmetrically dimethylated. Compensatory effects, or so-called substrate scavenging by type II PRMTs in the absence of type I PRMT activity has been previously demonstrated using PRMT knockout and knockdown cell lines to observe subsequent monomethyl arginine (MMA), asymmetric dimethylarginine (ADMA), and symmetric dimethylarginine (SDMA) levels (Dhar et al., 2013; Stouth et al., 2017). As mentioned before, this difference in histone methylation state might alter the chromatin remodeling process in a way that proves beneficial in the presence of toxic polyGR and polyPR by protective stress response mechanisms (Figure 4B).

As previously mentioned, the results of our studies suggest that polyGR and polyPR are themselves substrates for type I PRMTs (Premasiri et al., 2020). In the context of chromatin remodeling, this could manifest as PRMTs methylating aberrant polyGR and polyPR instead of histone arginine residues and important related proteins. In the absence of normal histone arginine methylation activity, the transcriptional initiation, DNA damage repair, and cell signaling controlled by this methylation would be limited or irregular, making it difficult for the cell to cope with stress. In theory, this phenomenon could contribute to additive or synergistic pathogenicity, as toxic polyGR and polyPR would be inducing cellular stress while limiting the cellular mechanisms in place to cope with that stress. Our results showing that NSC-34 cell toxicity produced by synthetic ADMe-GR_15_ or ADMe-PR_15_ challenge was not significantly abrogated by administration of a small molecule type I PRMT inhibitor to bolster the feasibility of this hypothesis. Because synthetic ADMe-GR/PR constructs were methylated before introduction to the cell, inhibition of type I PRMT activity would provide a limited rescue effect, as it can no longer inhibit the methylation of the aberrant GR or PR substrates. Instead, the PRMT activity that is regulating transcription, DNA damage repair, and cell signaling is inhibited—leaving the more potently toxic ADMe-GR/PR to disrupt cellular homeostasis (Figure 4C).

### Splicing

**Figure 5:**
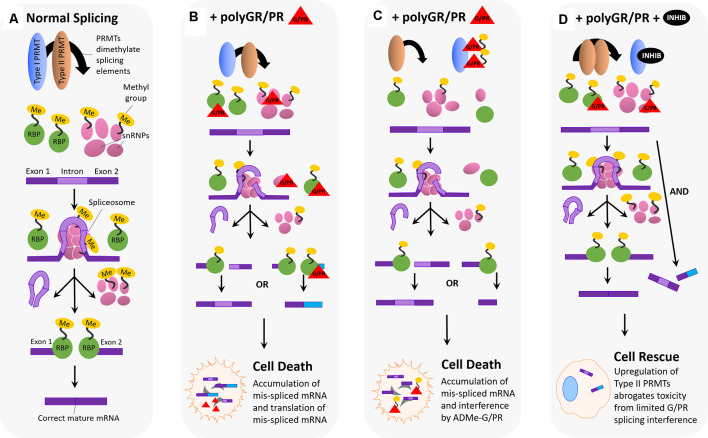
Hypothesized impact of polyGR/PR on splicing dynamics and rescue by PRMT inhibition. **(A)** Normal splicing activity and spliceosome assembly: for normal splicing activity to occur, type I and type II PRMTs are both involved in recruiting splicing elements to the RNA splice site, and this recruitment occurs *via* either asymmetric (Type I) or symmetric (Type II) dimethylation of arginine residues on RNA binding proteins (RBPs), small nuclear ribonucleoproteins (snRNPs), splicing factors, and other elements. Five snRNPs self-associate, as the spliceosome, at the site of an intron on premature mRNA, splicing the intron region out and discarding it. Following this step, the spliceosome disassembles, and snRNPs disassociate from the mRNA. RNA binding proteins then associate and join exon pieces to form the correct mature mRNA sequence. **(B)** Pathological scenario 1: splicing machinery recruited to the splice site by Type I and II PRMTs encounter GR and PR, which associate and interfere with their splicing activity. One possibility is that GR/PR association with snRNPs could result in incorrect spliceosome assembly, and thus incorrect intron excision, leading to mis-spliced mature mRNA containing intron fragments or missing exon fragments. Another possibility is that GR/PR association with RBPs could lead to incorrect exon selection during the splicing process, resulting in a mis-splicing event producing incorrect mature mRNAs. The following accumulation of mis-spliced mRNA, and translation of mis-spliced mRNA, could lead to cell death. **(C)** Pathological scenario 2: PolyGR/PR act as a sink recruiting Type I PRMTs, reducing asymmetric dimethylation and possibly increasing the proportion of symmetrically dimethylated splicing machinery. Type I PRMTs may instead asymmetrically dimethylate GR/PR to form ADMe-GR/PR, which may interfere with structures and proteins throughout the cell. Limited splicing machinery recruitment may result in impaired splicing with mature mRNA containing intron fragments or missing exon fragments. The accumulation of mis-spliced mRNA, ADMe-GR/PR, and the translation of mis-spliced mRNA could all lead to cell death. **(D)** Rescue scenario: with the administration of a type I PRMT inhibitor, it is possible that a robust compensatory upregulation of Type II PRMT activity is induced, resulting in more normalized levels of splicing activity such that limited interference by unmethylated GR/PR is not enough to produce detectable cytotoxicity.

RNA splicing, the process of removing intron sequences from pre-mRNA to produce and connect a final exon sequence (mature mRNA), is a cellular process that is integral to gene expression (Clancy, 2008; Figure 5A). The splicing process is catalyzed by the spliceosome, an RNP complex comprised of five snRNPs and many RNA processing proteins, such as RBPs (Will and Lührmann, 2011). Many proteins and splicing factors associated with the spliceosome are influenced by PRMT activity. PRMT1 has been shown to regulate the localization and function of several RBPs, including hnRNPA2 (Nichols et al., 2000), Sam68 (Côté et al., 2002), FUS/TLS (Tradewell et al., 2012), RNA binding motif protein 15 (RBM15; Zhang et al., 2015), and hnRNPA1 (Wall and Lewis, 2017). RBPs are capable of controlling splice site choice, as well as defining which exons are included in resulting mature mRNA (Yee et al., 2019), meaning that PRMT1 regulation of the localization and function of these proteins could affect splicing fidelity. Similarly, PRMT4/CARM1 is linked to splicing through its regulation of several RNA binding proteins (Cheng et al., 2007; Larsen et al., 2016). Most importantly, PRMT4 has been shown to asymmetrically dimethylate arginine residues on the transcription elongation regulator 1 (CA150/TCERG1), as well as splicing factor 3B subunit 4 (SAP49) and the U1 small nuclear ribonucleoprotein complex (U1 snRNP C) in the nucleus (Cheng et al., 2007). PRMT4 has also been shown to methylate Sm proteins in the Tudor domain of SMN, the *SMA* gene product (Cheng et al., 2007). The type II PRMTs, PRMT5, and PRMT9, also influence splicing activity. PRMT9 symmetrically dimethylates spliceosome-associated protein 145 (SF3B2), a component of the U2snRNP for the Tudor domain of SMN in the cytoplasm (Hadjikyriacou et al., 2015; Yang et al., 2015). PRMT5 plays a key role in ensuring snRNP maturation and splicing fidelity, methylating several RBPs (Meister et al., 2001; Meister and Fischer, 2002), and symmetrically dimethylating three out of seven Sm proteins (Friesen et al., 2001a, b; Meister et al., 2001; Meister and Fischer, 2002; Zhang et al., 2008).

Arginine-rich dipeptide repeat proteins polyGR and polyPR have also been shown to influence splicing activity and fidelity. PolyGR and polyPR pathogenically interact with proteins harboring LCDs, such as RBPs, which associate with the spliceosome (Lee et al., 2016; Lin et al., 2016). LCD proteins normally mediate the assembly of membrane-less organelles, including nuclear speckle domains, which are the site of splicing component localization (Li et al., 2013; Ramaswami et al., 2013; Taylor et al., 2016). Relatedly, it is hypothesized that mis-splicing in C9-ALS cells could be caused by polyGR and polyPR disruption of nuclear speckles and pathological interaction with essential splicing RBPs (Yin et al., 2017). One study observed roughly 5,000 mis-splicing events after challenging astrocytes with polyPR, suggesting that polyPR, either directly or indirectly, interacts with spliceosome components to decrease splicing fidelity (Kwon et al., 2014). Another study showed that polyGR and polyPR blocked spliceosome assembly and splicing activity in nuclear extracts, as well as demonstrated polyGR and polyPR association with U2snRNP (Yin et al., 2017). This study indicated that in C9 patient iPSC-derived motor neurons (iPSC-MNs), the U2snRNP is depleted from nuclear speckle domains and mislocalized to the cytoplasm. This effect could result from polyGR and polyPR influence, as shown in an assay that demonstrated U2snRNP mislocalization to the cytoplasm in HeLa cells following polyPR challenge (Yin et al., 2017). Gene ontology (GO) and gene set enrichment analysis (GSEA) analyses in the same study indicated that polyGR and polyPR interference with U2snRNP caused as much as 44% of the mis-spliced cassette exons in C9 patient brains (Yin et al., 2017). This evidence reinforces the idea that polyGR and polyPR can induce spliceosome dysfunction through interaction with a major snRNP to cause mis-splicing events.

This body of research, illustrating the interaction of PRMTs with key spliceosome components, in conjunction with that illustrating pathological interaction of polyGR and polyPR DRPs (Figure 5B) with these same spliceosome components to decrease splicing fidelity, raises important questions about the interaction of these two phenomena in C9-ALS. As previously mentioned, in our study, the application of small molecule type I PRMT inhibitors were capable of dose-dependently abrogating cytotoxicity associated with 24 h polyGR or polyPR challenge in NSC-34 cells (Premasiri et al., 2020). One possible explanation for the observed rescue effect could be that inhibiting type I PRMTs decreases asymmetric dimethylation of arginine residues on RBPs and other splicing proteins, in a way that is beneficial to the cell in the presence of polyGR and polyPR. Suppressed type I PRMT activity would not halt splicing activity entirely, considering symmetric dimethylation of splicing proteins by the type II PRMTs, PRMT5, and PRMT9, would not be decreased by type I PRMT inhibition. Instead, type I PRMT activity suppression would limit sequestration of those RBPs and snRNPs that are methylated by type I PRMTs to the spliceosome, potentially limiting the chance for pathological polyGR and polyPR interaction with those key proteins. This scenario could lead to increased type II PRMT activity at the spliceosome to compensate for decreased type I PRMT activity, leading to the symmetric dimethylation of spliceosome proteins that are typically asymmetrically dimethylated (Figure 5D). As previously mentioned, this *substrate scavenging* by type II PRMTs in the absence of type I PRMT activity has been previously demonstrated using knockout and knockdown cell lines of various PRMTs (Dhar et al., 2013; Stouth et al., 2017). A change in the methylation state of spliceosome proteins could also affect their activity in a way that is protective in the presence of polyGR and polyPR.

Again, the results of our study also raise the possibility that polyGR and polyPR are themselves substrates for type I PRMTs (Premasiri et al., 2020). In the context of RNA splicing, this could manifest as PRMTs methylating aberrant polyGR and polyPR instead of key splicing proteins, resulting in mislocalization of these proteins such that they disrupt the activity of splicing proteins to promote more frequent mis-splicing events (Figure 5C) and/or otherwise disrupt cellular homeostasis. In the absence of normal splicing activity, the cell would be less able to generate the mature mRNAs necessary to translate key proteins needed to function normally or to cope with cellular stress. Also, the generation of mis-spliced, aberrant mRNAs could enhance cellular stress. This process could manifest as synergistic pathogenicity. Further, this toxicity would be compounded with the possible enhanced toxicity of ADMe-polyGR and ADMe-polyPR (Premasiri et al., 2020). Thus, the rescue effect we see in our assay upon administering type I PRMT inhibitors to NSC-34 cells that have been challenged with polyGR and polyPR could reflect decreased methylation of polyGR and polyPR by type I PRMTs in a way that protects the cell from splicing protein mislocalization, splicing infidelity, and added cellular stress.

## Conclusion

Methylation of arginine affects cellular functions in ways both subtle and profound; and in ways that are now only beginning to be understood. It should not be surprising that the modification of the most basic and positively charged amino acid should be impactful. Indeed, as discussed in this article, the methylation of arginine residues in various proteins changes physicochemical properties that drive probabilities of multiple biochemical interactions. For example, methylation of arginine residues in RBPs can reduce their propensity to associate with RNAs. Also, methylation of arginine residues can decrease propensities of proteins with arginine motifs to associate with other proteins. These alterations of physicochemical properties have higher-order cellular implications; for example, potentially altering RNA splicing and influencing LLPS dynamics which are important in the formation and disassembly of stress granules and other membraneless organelles.

The centrality of RBP function, LLPS separation dynamics, and membraneless organelle formation to the emerging understanding of ALS/FTD pathogenesis cannot be ignored. Similarly, recent findings implicating arginine-rich dipeptide repeat proteins, polyGR and polyPR, as drivers of C9orf72 G4C2_n_ mediated neurodegeneration should not be ignored.

Here, in the context of our findings that Type I PRMT inhibitors can be protective against polyGR/PR toxicity, we offer hypotheses for how arginine methylation of polyGR/PR might contribute to C9orf72 G4C2_n_-mediated neurodegenerative cytotoxicity. Further, we posit that the presence of polyGR/PR may act as a sink for PRMT activity, impeding essential post-translational modifications of RBPs or proteins like SmD1 and SmD3 which are more likely to associate with SMN and other Tudor domain-containing proteins following arginine methylation.

Much of current literature focused on the roles of methylation on RBP function, LLPS separation dynamics, and membraneless organelle formation and disassembly parse methylation states into two categories: methylated or unmethylated. However, very little research up to this point has delineated the specific impacts of monomethylation, asymmetric dimethylation, or symmetric dimethylation of arginine residues on these biological functions. Our data in cell-based models of polyGR/PR toxicity suggest that ADMe plays a toxicity-enhancing role. Because ADMe and SDMe are mutually exclusive, our findings are consistent with the clinical observation by Gittings et al. (2020) demonstrating that SDMe-GR staining correlates with later disease onset and slower disease progression in people carrying C9orf72 mutations– indicating that symmetric dimethylation may be protective. However, cell-based toxicity assays and LLPS results by the same laboratory indicated that both ADMe-GR and SDMe-GR exhibited reduced phase separation and cytotoxicity. Unlike our assay system, which applied polyGR 15 mers with every arginine residue methylated, Gittings et al. (2020) applied polyGR 20 mers with every odd arginine residue methylated. These disparate results derived from experimental systems employing such subtly different reagents indicate that we are confronted with an underdeveloped understanding of very sensitive biological systems. Careful follow-up experiments are warranted.

Stress granule biology, RNA splicing, and LLPS are all actively being explored as biology domains for therapeutic intervention in ALS/FTD, but, up to now, PRMTs have not been studied as therapeutic targets for ALS. PRMTs are important players in each of those domains. There are at least six Type I PRMTs, each with evidence of tissue and cell-type-specific expression, and each with some evidence of substrate specificity. Furthermore, PRMT inhibitors are in clinical development for other disease indications, indicating a general proof-of-concept that they are viable targets for human therapeutic intervention. Given these facts, while acknowledging that this research area remains in its infancy, we feel there is potential for the discovery and development of specific, safe, and effective therapeutics targeting PRMTs for C9orf72 G4C2_n_-mediated ALS/FTD.

## Data Availability Statement

The original contributions presented in the study are included in the article, further inquiries can be directed to the corresponding author.

## Author Contributions

AG, AP, and FV are equal contributors to this work and approve of its publication. All authors contributed to the article and approved the submitted version.

## Conflict of Interest

The authors declare that the research was conducted in the absence of any commercial or financial relationships that could be construed as a potential conflict of interest.
